# Vitamin D manipulates miR-181c, miR-20b and miR-15a in human umbilical vein endothelial cells exposed to a diabetic-like environment

**DOI:** 10.1186/1475-2840-13-8

**Published:** 2014-01-07

**Authors:** Tali Zitman-Gal, Janice Green, Metsada Pasmanik-Chor, Eliezer Golan, Jacques Bernheim, Sydney Benchetrit

**Affiliations:** 1 Renal Physiology Laboratory, Department of Nephrology and Hypertension, Meir Medical Center, Kfar Saba 44281, Israel; 2 Bioinformatics Unit, The G. S. Wise Faculty of Life Sciences, Tel Aviv University, Tel Aviv, Israel; 3 Sackler Faculty of Medicine, Tel Aviv University, Tel Aviv, Israel

**Keywords:** microRNA, Human umbilical vein endothelial cells, HUVEC, Diabetes, Calcitriol, Vitamin D, Endothelial cells, Diabetic environment

## Abstract

**Background:**

High blood and tissue concentrations of glucose and advanced glycation end-products are believed to play an important role in the development of vascular complications in patients with diabetes mellitus (DM) and chronic kidney disease. MicroRNAs (miRNA) are non-coding RNAs that regulate gene expression in a sequence specific manner. MiRNA are involved in various biological processes and become novel biomarkers, modulators and therapeutic targets for diseases such as cancer, atherosclerosis, and DM. Calcitriol (the active form of vitamin D) may inhibit endothelial proliferation, blunt angiogenesis, and be a cardioprotective agent. Calcitriol deficiency is a risk factor for DM and hypertension. The aim of this project was to study the miRNA microarray expression changes in human umbilical vein endothelial cells (HUVEC) treated in a diabetic-like environment with the addition of calcitriol.

**Methods:**

HUVEC were treated for 24 h with 200 μg/ml human serum albumin (HSA) and 100 mg/dl glucose (control group) or 200 μg/ml AGE-HSA, and 250 mg/dl glucose (diabetic-like environment), and physiological concentrations (10^-10^ mol/l) of calcitriol. miRNA microarray analysis and real time PCR to validate the miRNA expression profile and mRNA target gene expression were carried out.

**Results:**

Compared to control, 31 mature human miRNA were differentially expressed in the presence of a diabetic-like environment. Addition of physiological concentrations of calcitriol revealed 39 differentially expressed mature human miRNA. MiR-181c, miR-15a, miR-20b, miR-411, miR-659, miR-126 and miR-510 were selected for further analysis because they are known to be modified in DM and in other biological disorders. The predicted targets of these miRNA (such as KLF6, KLF9, KLF10, TXNIP and IL8) correspond to molecular and biological processes such as immune and defense responses, signal transduction and regulation of RNA.

**Conclusion:**

This study identified novel miRNA in the field of diabetic vasculopathy and might provide new information about the effect of vitamin D on gene regulation induced by a diabetic-like environment. New gene targets that are part of the molecular mechanism and the therapeutic treatment in diabetic vasculopathy are highlighted.

## Introduction

MicroRNAs (miRNA) are non-coding RNA species of approximately 22 nucleotides that regulate gene expression in a sequence-specific manner. miRNA join to partial complementary sequences in the 3'UTRs of target mRNA of protein coding genes to specify translational repression and/or mRNA cleavage [1,2]. miRNA are involved in various biological processes and have become novel biomarkers, modulators and therapeutic targets for diseases such as cancer, heart disease, and diabetes [1,2]. Several studies investigated the effect of miRNA on the development of diabetes and its complications, including endothelial and vascular smooth muscle cell dysfunction, diabetic cardiomyopathy and diabetic nephropathy, and demonstrated involvement of specific types of miRNA, which regulate a broad range of inflammatory genes [3-5].

Diabetes mellitus (DM) is associated with endothelial dysfunction including changes in barrier function and homeostasis, reduced vasodilator response, inflammatory activation, increased plasma levels of endothelial products, and angiogenesis, all of which are associated with a greater incidence and severity of cardiovascular diseases [6-10]. Hyperglycemia is thought to affect endothelial function, increasing stiffness of the peripheral arteries and arterioles partly due to reduced nitric oxide production [8,10]. Elevated concentrations of environmental glucose initiate events that stimulate extracellular and intracellular formation of advanced glycation end products (AGEs) [10-12]. The increased formation of AGEs plays a relevant role in the development of vascular and renal-related complications seen in DM, chronic kidney disease (CKD), and aging [11,12]. Previous studies performed in our laboratory have demonstrated that AGEs stimulate the endothelial expression of AGE receptor (RAGE) and interleukin 6 (IL6), and depress the eNOS mRNA expression and eNOS enzymatic activity [13,14], while calcitriol blunted the deleterious effect of AGEs in this *in vitro* model [14].

Our previous studies in endothelial and vascular smooth muscle cells using a high throughput microarray approach [15-17], showed that diabetic-like conditions (250 mg/dl glucose and AGEs at a concentration similar to that found in the blood of diabetic patients) induced significantly elevated expressions of cellular and metabolic processes, including endothelial inflammatory-related genes through the NFκB pathway and stimulated the production of thioredoxin interacting protein (TXNIP). Calcitriol (1,25 dihydroxycholecalciferol), the active form of vitamin D that controls calcium homeostasis, hormonal secretions, and cell proliferation and differentiation, was found to have a protective effect on the development of renal or cardiovascular disorders in stimulated endothelial cells in vitro and in vivo [18,19]. Vitamin D deficiency is a risk factor for DM and hypertension [20]. Our previous studies showed that adding calcitriol to endothelial and vascular smooth muscle cells significantly down-regulated the inflammatory response of gene and protein expression involved in the NFκB signal transduction pathway [16,17]. In this study we evaluated the expression of miRNA and their predicted gene targets in human umbilical vein cord endothelial cells (HUVEC) exposed to a diabetic like environment and the effect of calcitriol on their expression.

## Materials and methods

### Cell culture and incubation

HUVEC were isolated from umbilical cords obtained from the maternity unit at Meir Medical Center, Kfar Saba, Israel [13]. The Ethical Review Committee approved the study and the parturient provided written informed consent. HUVEC were identified by their typical cobblestone morphology and by immunostaining for von Willebrand factor. HUVEC were grown in M-199 medium supplemented with 20% FCS, 100 U/ml penicillin, 100 μ/ml streptomycin (Biological Industries, Bet Haemek, Israel), 5 U/ml heparin, and 25 μ/ml endothelial mitogen (Biomedical Technologies Inc., Stoughton, MA, USA). Confluent cultures of HUVEC were used for experiments at passages 3–4. The cells were stimulated in media containing 200 μg/μl HSA and 100 mg/dl glucose (control group) or 200 μg/μl AGE-HSA and 250 mg/dl glucose (diabetic-like environment) for 24 h. Calcitriol 10^-10^ mol/l (PH&T SpA, Milan, Italy), corresponding to physiological blood concentrations, [16,17] was added to cells 1 h after having started the stimulation with AGE-HSA and glucose for an additional 23 h.

### miRNA microarray analysis

miRNA were extracted using the mirVANA™ RT-PCR miRNA isolation and detection kit (Ambion, Austin, Texas, USA) according to manufacturer’s instructions. RNA quantity and quality were determined using Nanodrop (Thermo Fisher Scientific, Inc, Wilmington, DE, USA). Affymetrix GeneChip® miRNA 2.0 arrays were used for genome-wide miRNA expression analysis (15,644 probe sets for 131 organisms, including 1,105 human miRNA, 1,105 human pre-miRNA and 2,302 human small nucleolar RNAs) according to the instruction manual, as described in URL 1 (Affymetrix, Santa Clara, CA, USA). Three biological repeats were used for each treatment.

### Bioinformatics analysis

Microarray analysis was performed on CEL files using Partek® Genomics Suite™ (Partek GS, Partek Inc., MO, USA; Quantile normalization was performed by the robust multi-average method (RMA). Batch effect removal was applied for the different samples to remove individual variations, followed by one-way analysis of variance (ANOVA). Human miRNA that were differentially expressed (p < 0.05; fold-change cutoff 1.5) were obtained. Each relevant miRNA was analyzed using TargetScan, which uses a computational algorithm to predict biological gene targets of miRNA. David and WebGestalt databases (http://david.abcc.ncifcrf.gov and http://bioinfo.vanderbilt.edu/webgestalt, respectively) were used for functional annotations of the target gene lists. The String database (http://string-db.org) was used to predict the miR-target-interactions including direct (physical) and indirect (functional) associations. Enrichment of GO biological processes with FDR correction was used.

### miRNA – real time PCR

miRNA real time PCR was performed on 2 additional biological repeats (total of 5 repeats) using TaqMan specific Small RNA primer and probe sets for miR-181c, miR-411, miR-659, miR-510, miR-126, miR-15a and miR-20b (Applied Biosystems, Inc., Foster City, CA, USA). miRNA expression is represented relative to the expression of the internal control U6-snRNA. Data were analyzed using the 2^-ΔΔCt^ method.

### RNA extraction and real-time PCR

Total RNA was extracted from HUVEC using mirVANA™ RT-PCR isolation and detection kit (Ambion, Austin, Texas, USA) and RNeasy Mini Kit, including DNase digestion with RNase-free DNase set (Qiagen, Valencia, CA, USA) according to manufacturer's instructions. RNA integrity was assessed using NanoDrop (Thermo Fisher Scientific, Inc, Wilmington, DE, USA). RNA (1 μg) was then reverse transcribed into single-strand DNA using the High Capacity cDNA Reverse Transcription Kit (Applied Biosystems, Inc, Foster City, CA, USA), according to manufacturer's instructions. TaqMan real-time PCR amplification was used with gene specific primer for Kruppel-like Factor 6 (KLF6), KLF9, KLF10 using glucuronidase, beta (GUSB) as control genes. Data were analyzed using the 2^-ΔΔCt^ method.

### Statistical analysis

All data are expressed as mean ± standard deviation (SD). Friedman and repeated measures with Bonferroni multiple comparisons were used to evaluate the effect of the different treatments on the expression of selected miRNA. Student *t*-test and Mann–Whitney non-parametric test were used to evaluate the different treatments on the expression of selected target mRNA genes. P-values of 0.05 or less were considered significant.

## Results

### Differentially expressed miRNA

**Figure 1:**
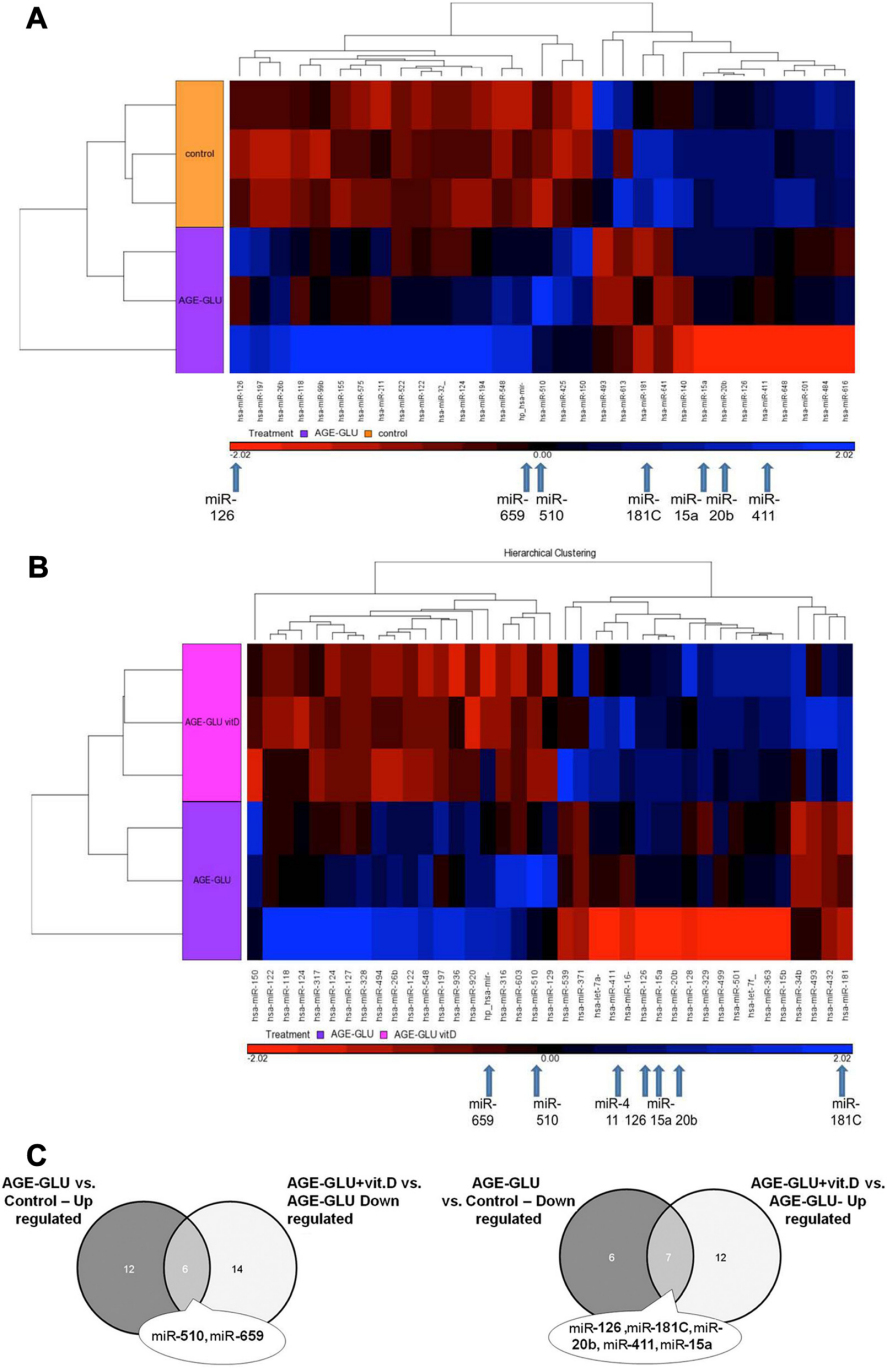
**Hierarchical clustering of miRNA expression in HUVEC.** Seven focal miRNA are indicated with arrows at the bottom of the clusters **(A, B)** and on the VENN diagram **(C)**. Clusters represent treatment with **(A)** AGE-HSA and glucose 250 mg/dl (AGE-GLU) compared to control (HSA and 100 mg/dl glucose concentration); **(B)** AGE-HSA, glucose 250 mg/dl and 10^-10^ mol/l calcitriol (AGE-GLU + vit. D) compared to AGE-HSA and glucose 250 mg/dl (AGE-GLU); **(C)** Comparison between differentially expressed miRNA lists.

MiRNA expression in cultured HUVEC exposed to a diabetic-like environment revealed 31 mature human miRNA that were significantly changed (p < 0.05, fold change cut-off 1.5), of which 18 were up-regulated and 13 were down-regulated (Figure 1A).

**Figure 2:**
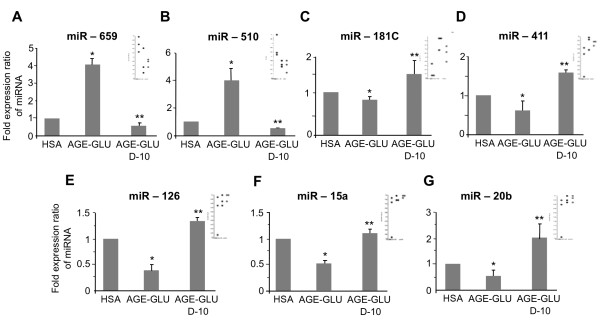
**Validation of miRNA expression results by real time PCR*.*** HUVEC were incubated for 24 h with HSA (200 μg/ml), AGE-HSA (200 μg/ml) and glucose (250 mg/dl). In addition, 10^-10^ mol/l calcitriol was given to the cells 1 h after stimulation for an additional 23 h. The miRNA set that included **(A)** miR-659, **(B)** miR-510, **(C)** miR-181C, **(D)** miR-411, **(E)** miR-126, **(F)** miR-15a, and **(G)** miR-20b was validated using real time PCR. Insets show miRNA microarray expression results. Data are expressed as mean ± SD of 4–5 independent experiments. *P < 0.05 compared to control group-HSA. **P < 0.05 compared with AGE-glucose.

The addition of calcitriol (10^-10^ mol/l) to cultured HUVEC exposed to a diabetic-like environment revealed 39 mature human miRNA that were significantly changed (p < 0.05 and fold-change cut-off 1.5), of which 19 miRNA were up-regulated and 20 were down-regulated (Figure 1B). Table 1 presents a partial list of selected miRNA (from the differential mature human miRNA) that were chosen according to their p-value, their fold changes or their physiological expression. From the miRNA list presented in Table 1 and from the corresponding Venn diagram (Figure 1C), we validated several miRNA (marked in bold in Table 1) that are known to be modified in a diabetic environment (miR-510, miR-15a, miR-20b, miR-126, and miR-181C). miR-659 and miR-411 were validated because in the microarray analysis they were significantly changed after the addition of calcitriol. Each miRNA was normalized to that of the U6-snRNA, which was used as a reference. A total of 5 biological repeats (5 different umbilical cords) were used for the validation to ensure that the variations observed were biological. The miRNA expression patterns (Figure 2, A-G) were consistent between the microarray (insets) and real-time PCR validations.

**Table 1:** Summary of miRNA in HUVEC exposed to a diabetic environment with and without calcitrol

**miRNA ID**	**Diabetic environment**^**a**^		**Diabetic environment and calcitrol**	
	**Fold change**	**p-value**	**Fold change**	**p-value**
hsa-mir-600	1.53	0.014	-1.35	0.036
hsa-mir-10b	1.49	0.01	-1.31	0.032
hsa-mir-640	1.31	0.006	-1.35	0.004
**hsa-miR-510**	1.35	0.018	-1.49	0.03
**hsa-miR-659**	1.37	0.13	1.99	0.0005
hsa-mir-320e	1.88	0.002	-1.12	0.19
miR-425-star	1.8	0.01	-1.48	0.034
**hsa-miR-181c**	-1.15	0.14	3.26	0.01
**hsa-miR-411**	-1.57	0.015	2.33	0.002
hsa-miR-133b	-1.73	0.002	1.19	0.056
hsa-miR-613	-1.91	0.005	1.38	0.036
hsa-miR-335	-1.71	0.037	1.97	0.019
hsa-miR-642	-1.48	0.006	1.23	0.036
hsa-miR-670	-1.41	0.026	1.53	0.014
**hsa-miR-20b**	-2.62	0.072	2.86	0.09
**hsa-miR-15a**	-4.54	0.07	3.69	0.11
**hsa-miR-126**	-1.76	0.07	1.55	0.14

^a^200 μg/ml AGE-HSA and 250 mg/dl glucose vs. control (200 μg/μl HSA and 100 mg/dl glucose).

### Gene target and pathway analysis of miR-181C, miR-15a and miR-20b

**Figure 3:**
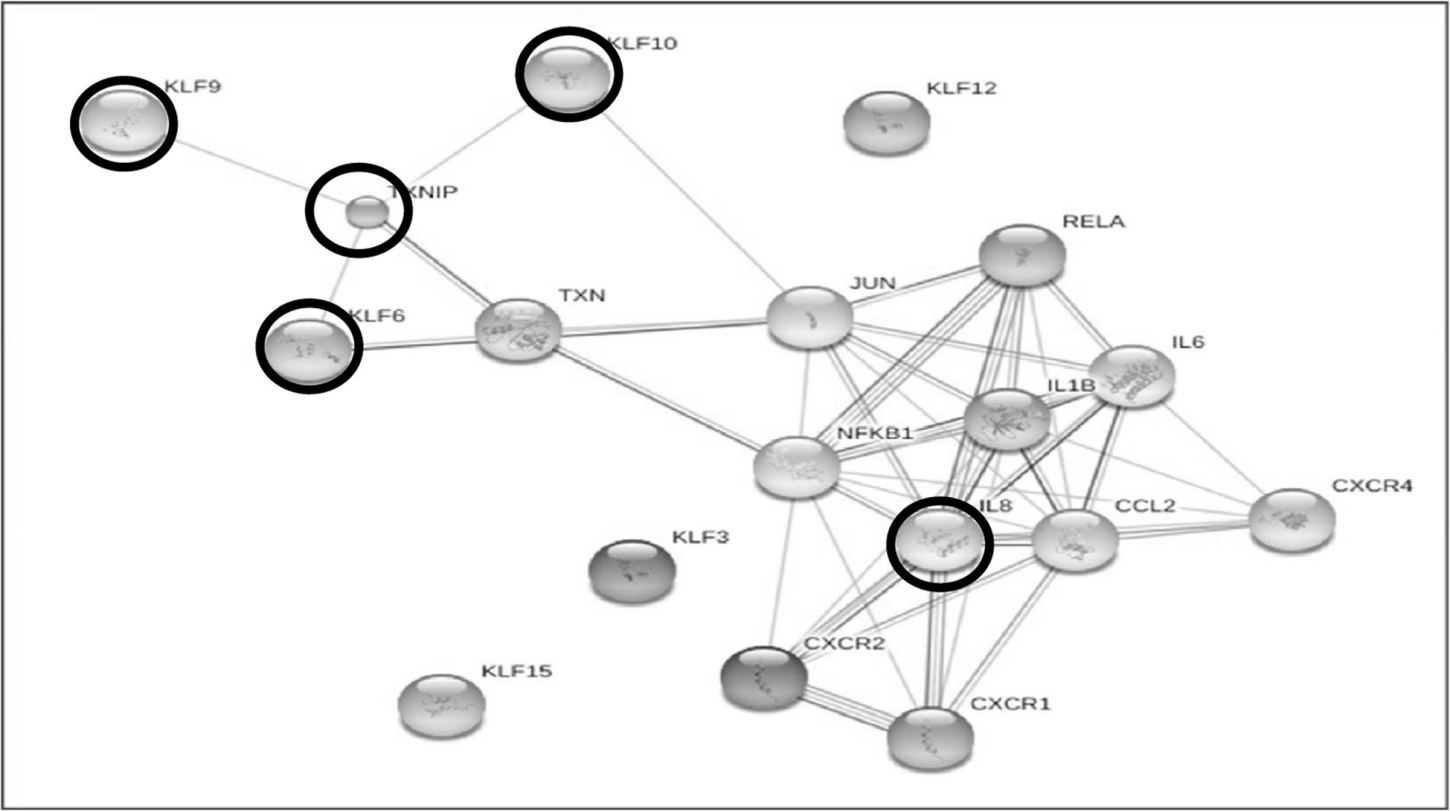
**Predicted miRNA-target protein biological interactions, including direct and indirect associations.** The predicted gene targets were found to correspond to biological processes such as immune and defense responses, signal transduction and regulation of RNA and primary metabolic processes. The genes that were further analyzed and discussed, KLF6, KLF9, KLF10, TXNIP and IL8 are marked with a ring. These gene targets were chosen because they take part in cellular growth, differentiation, proliferation, apoptosis and angiogenesis, as well as in regulating endothelial cell metabolism, growth, and inflammation.

**Figure 4:**
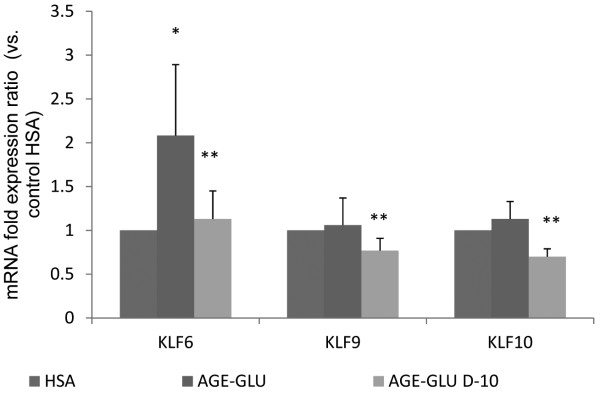
**Effect of calcitriol on target genes KLF6, KLF9 and KLF10 mRNA expression in HUVEC stimulated with a diabetic-like environment.** HUVEC were incubated for 24 h with HSA (200 μg/ml), AGE-HSA (200 μg/ml) and glucose (250 mg/dl). In addition, 10^-10^ mol/l calcitriol was given to the cells 1 h after stimulation for an additional 23 h. KLF6, KLF9 and KLF10 mRNA expression were analyzed by real-time PCR and normalized to the GUSB. Data are expressed as mean ± SD of 4–5 independent experiments. *p < 0.05 compared to control group-HSA; **p < 0.05 compared with AGE-Glucose.

MiRNA are known to regulate many target genes and consequently can modulate different signaling pathways. We focused on three differentially expressed miRNA: miR-181C, miR-15a and miR-20b, which were found to be down-regulated in a diabetic-like environment and up-regulated after the addition of calcitriol. They were chosen for further investigation because their gene targets play a key role in endothelial cell function, which is relevant to our research (Table 2). We found that genes targets from the Kruppel-like family, which are transcription factors that play key regulatory roles in cellular growth, differentiation, proliferation, apoptosis and angiogenesis [20,21], take part as putative targets for miR-181C and miR-20b. TXNIP, a pro-apoptotic protein, which is known to regulate endothelial cell metabolism, growth, and inflammation [22,23] and IL8, an inflammatory-related protein [24] are putative targets of miR-20b and miR-15a. Based on these target genes, using the String database, we predicted the miRNA-target protein interactions including direct (physical) and indirect (functional) associations. Based on these results, we found that the predicted gene targets correspond to biological processes such as immune and defense responses, signal transduction and regulation of RNA and primary metabolic processes (Figure 3). We decided to further analyze KLF6, KLF9 and KLF10 and their expression patterns. Using real time PCR analysis, we found that the expression level of KLF6 was significantly up-regulated in a diabetic-like environment (Figure 4). KLF9 and KLF10 were also up-regulated, but not significantly. The addition of calcitriol significantly down-regulated KLF6, KLF9 and KLF10 mRNA expression (Figure 4). TXNIP and IL8 expression patterns, as previously described, were significantly up-regulated in a diabetic-like environment, whereas the addition of calcitriol significantly down-regulated IL8 [14,15].

**Table 2:** Putative target genes of the selected miRNA (Target scan analysis)

	**miR-181c**	**miR-20b**	**miR-15a**
**KLF6**	**KLF6**		
**KLF15**	**KLF15**		
**KLF3**		**KLF3**	
**KLF9**	**KLF9**		
**KLF10**	**KLF10**	**KLF10**	
**KLF12**		**KLF12**	
**IL8**		**IL8**	
**TXNIP**		**TXNIP**	**TXNIP**

## Discussion

### Endothelial cell, diabetic environment and vitamin D

The regulation of miRNA that are released in the endothelium in response to a diabetic-like environment and vitamin D might provide important insights to the events that play a major role in the development of vascular complications. Our previous studies showed that diabetic-like conditions characterized by mildly elevated glucose concentrations (250 mg/dl) and AGEs in an HUVEC culture model, were found to stimulate endothelial TXNIP system activity, as well as expression of the inflammatory related proteins IL6, IL8, RAGE, and NFκB [15]. Adding physiological concentrations of calcitriol (10^-10^ mol/l) to this in vitro model had a beneficial effect on the endothelial expression of pro-inflammatory parameters, probably through the NFκB signal transduction pathway [16]. Vitamin D was found to play an important role in renal, endothelial, and cardiovascular protection and to have antitumor activity [18-20]. In this study, we used a miRNA microarray to obtain expression profiles of miRNA in cultured HUVEC that were exposed to a diabetic-like environment in order to mimic a clinical model of DM in vitro. We demonstrated that stimulation of vitamin D in this type of environment has a beneficial effect on the endothelial expression of miRNA profile and their target genes.

### Differentially expressed miRNA

We identified a list of miRNA that were differentially expressed following the various manipulations. As mentioned previously, we focused on selected miRNAs that are known to be modified in a diabetic-like environment or that were highly changed after the addition of calcitriol. MiR-510 and miR-659 were over-expressed under diabetic-like conditions and decreased after calcitriol was added. MiR-126, miR-411, miR-20b, miR-15a and miR-181c were down-regulated under diabetic conditions and over-expressed after calcitriol was added.

MiR-510 was first identified in association with irritable bowel syndrome; its co-expression was involved in the regulation of 5-HT_3_ receptors in colonic enterocytes [23]. Recently, Hezova et al. [24] demonstrated that miR-510 expression was elevated in regulatory T cells of diabetic patients. To the best of our knowledge, our findings are the first demonstration of miR-510 involvement in HUVEC exposed to a diabetic-like environment with significant changes induced by calcitriol. Manipulation of miR-510 may represent a beneficial effect of calcitriol if we consider the possible deleterious effect of increased miR-510 in DM. MiR-659 is not described in the database as being connected to a diabetic-like environment and appears to be novel in genomic control in the presence of diabetic-like conditions affected by vitamin D. Further investigation of the effect of the miRNA on their predicted target genes is warranted.

MiR-126 is highly enriched in endothelial cells and is involved in vascular integrity, angiogenesis and wound repair [25,26]. Moreover, loss of plasma miR-126 is consistently associated with diabetes [27,28]. Zampetaki et al. [27] found that high glucose concentrations (25 mmol/l equivalent to 500 mg/dl) significantly reduced miR-126 expression in endothelial apoptotic bodies and improved vascular growth factor (VEGF) signaling, leading to endothelial dysfunction [27,29]. In patients with DM, low plasma levels of miR-126 could be clinically relevant and contribute to VEGF resistance and endothelial dysfunction [30]. In this study, we also found in HUVEC a decreased expression of miR-126 exposed to a diabetic like environment and demonstrated that the addition of calcitriol elevated miR-126 expression, which can probably improve VEGF signaling and repair endothelial dysfunction.

Differential expression of miR-411 was reported in a miRNA profiling study of the hippocampus and the marginal division in a rat brain [30]. In addition, miR-411 was reported to be down-regulated in an autosomal dominant muscle disorder (facioscapulohumeral muscular dystrophy) suggesting that reduction of miR-411 might have a positive effect on muscle regeneration and promote myoblast maturation [31]. To our knowledge, no study has investigated the effect of diabetes and vitamin D on the function of miR-411 in endothelial cells. The relevance of its role in diabetes remains unknown and should be clarified in the future. The significant effect of vitamin D remains speculative at this stage.

### Selected miRNAs and their target genes

MiR-15a, miR-20b and miR-181C were found to be down-regulated in a diabetic-like environment and up-regulated after the addition of calcitriol; they were chosen for further investigation at the level of their gene targets, which have been shown to be involved in the modulation of endothelial function. MiR-15a, a cell growth suppressor, was evaluated in human cancer cells and found to have a pro-apoptotic role by activating caspase 3/7, which reduces cell viability [32]. In addition, miR-15a has been correlated with different pathophysiological events in the liver, which are also side-effects of anabolic steroids [33]. MiR-15a was down-regulated in the plasma of diabetic patients [27] and in β-cells exposed to high glucose (33 mM equivalent to 600 mg/dl) for long periods [34]. Mir-15a was also found to be up-regulated in endothelial cells and vascular smooth muscle cells after stimulation with KLF4, which indicate an option to suppress proliferative vascular disorders [35]. MiR-20b was down-regulated in the plasma of diabetic patients [27] and similarly expressed in normal and diabetic dermatological tissue, but was significantly different from that of diabetic wounds during the course of healing [36]. To our knowledge, no study has investigated the effect of diabetes and vitamin D on the function of miR-20b in endothelial cells.

Using pediatric cancer stem cells, Sanchez-Diaz et al. [37] showed that miR-181c regulate cell proliferation and the cell cycle, probably by affecting the Notch signaling pathway and the bone morphogenetic protein (BMP) pathway. Becker et al. [33] demonstrated that under the influence of anabolic steroids, miR-181c was down-regulated in bovine liver and might lead to uncontrolled proliferation in the liver.

To our knowledge, no study has investigated the effect of diabetes and vitamin D on the function of miR-181C in endothelial cells.

As mentioned above, these three miRNA that were down regulated in a diabetic environment were significantly up-regulated after addition of calcitriol. To the best of our knowledge, this is the first demonstration of the effect of calcitriol on the expression of these three miRNA under diabetic-like conditions in HUVEC.

In order to determine the potential genes involved in HUVEC exposed to a diabetic-like environment and calcitriol, we analyzed the predicted target genes of these 3 miRNA (miR-15a, miR-20b and miR-181c).

Seven predicted targets of the 3 deregulated miRNA were chosen for further analysis of the molecular pathways. In Figure 3, we observed that the target genes KLF9, KLF10, and KLF6 are directly and indirectly (via TXN, JUN and FOS) bound to TXNIP and to IL8 and correspond to immune, defense and metabolic biological processes. After calcitriol was added to the cells, we observed a down-regulation in KLF9, KLF10, KLF6, TXNIP and IL8 expression. These findings were negatively correlated with the miRNA expression patterns and therefore support their function.

Kruppel-like factors are members of the zinc finger family of transcription factors that regulate cellular differentiation and tissue development [38,39]. KLF9 is a transcriptional regulator that is highly expressed in the rat brain, kidney, lung, and testis and regulates uterine endometrial cell proliferation, adhesion and differentiation [40,41]. Panda et al. [42] showed that in Ishikawa cells (an endometrial adenocarcinoma cell line), miR-200c increased cell proliferation through KLF9 repression.

KLF10 plays a major role in mediating the effects of TGF-β through regulation of the Smad signaling pathway. It regulates gene transcription, inhibits cell proliferation, induces apoptosis and plays a role in activating the inflammatory response, including stress-induced inflammation, leading to increases in cardiovascular diseases, autoimmune abnormalities and DM [43]. Yang et al. [22] showed that KLF10 induced endothelial cells to facilitate a possible pathway for TGF-β and regulated COX-1, which affects platelet aggregation. Using microarray gene expression, we demonstrated that in vascular smooth muscle cells, KLF10 was up-regulated under diabetic conditions and down-regulated after calcitriol was added [17]. In this study, calcitriol had also a beneficial effect on KLF10 mRNA expression in HUVEC exposed to a diabetic environment.

KLF6 is one of the KLFs that are reportedly expressed in endothelial cells and is considered a damage-response factor [38,39,44]. KLF6 promotes tissue remodeling due to its ability to activate genes that are members of the TGF-β signaling pathway, which are implicated in vascular remodeling, tumor metastasis and apoptosis [39,43]. Qi et al. [45] demonstrated that KLF6 was induced by high glucose concentrations (30 mmol/l equivalent to 600 mg/dl) in human kidney cells and binds to the TXNIP promoter region. In addition, in an *in vivo* study in diabetic rats, they showed that KLF6 and PPAR-γ (localized downstream of KLF6) play a key role in the regulation of TXNIP expression in the development of diabetes mellitus. The KLF target genes that were analyzed in this study and were found to be modified by a diabetic-like environment and calcitriol might provide unique information on the function of these transcription factors in endothelial cells and may be the basis of attractive research in the future in the field of DM in animal models and humans.

TXNIP is stimulated by high glucose concentrations. It is well-demonstrated that it promotes oxidative stress and apoptosis in several types of cells, including endothelial cells [15,46,47]. In this study, we showed that TXNIP mRNA expression in cultured HUVEC was up-regulated following exposure to a diabetic-like environment, while no significant changes was observed after the addition of calcitriol. IL8 is produced in several tissues, as well as in endothelial cells upon infection, inflammation, ischemia and trauma [48]. We found that diabetic-like conditions stimulate the expression of this inflammation-related protein and that the addition of calcitriol had a beneficial effect on its expression.

### Vitamin D deficiency and supplementation

It has been well established that vitamin D deficiency is correlated with high body fat and glucose levels and decreased insulin sensitivity. It is also an independent cardiovascular risk factor that predicts poor cardiovascular outcomes [49-51]. Grineva et al. [49] showed that in women at late reproductive age (mean age 46.1 ± 4.5) from the North‒West region of Russia, low 25(OH)D levels were associated with obesity, increased plasma glucose levels after OGTT and insulin resistance. A significant correlation between fasting insulin, 2 h OGTT glucose, insulin levels and low 25(OH)D levels was found in overweight and obese subpopulations. The authors suggested that vitamin D supplementation could be effective in the prevention of obesity and insulin resistance [49]. The intervention between obesity, vitamin D and PTH is complex, suggesting possible effects at different levels. Alkharfy et al. [50] showed a beneficial effect of vitamin D supplementation in the metabolic profile of DM type 2 patients treated with insulin and oral hypoglycemic agents. The authors suggested that the effect of the medication could be mediated by an increase in HDL-cholesterol levels, particularly by vitamin D [50]. Al-Daghri et al. [51] showed an improvement in LDL, total cholesterol and in homeostasis model assessment of β-cell function (HOMA-β) among a Saudi DM type 2 population receiving vitamin D_3_ supplementation for 18 months. This effect was more pronounced in women than in men. Vitamin D supplementation was suboptimal, which could explain the absence of significant increase in HDL-cholesterol levels and the increase in HOMA-insulin resistance index. Moreover the absence of relevant information concerning the anti-diabetic drugs and the diet composition of the patients could have limited the impact of the results [51].

Ongoing studies should provide an answer to the still-debated question, whether vitamin D supplementation can positively influence cardiovascular outcomes. Our study showed that vitamin D can influence the pro-atherogenic and inflammatory response seen in DM and therefore, might contribute to a preventive and therapeutic effect. Moreover, recent study showed that vitamin D may improve endothelial function in cardiovascular diseases and could possibly have a role in vascular protection [52].

## Conclusions

In this study, we tried to mimic a diabetic-like environment *in vitro* and added vitamin D to simulate the replacement therapy frequently given to vitamin D deficient patients with DM. Vitamin D deficiency might have a negative effect on endothelial cell functions. Treating these patients to obtain normalization of their vitamin D reserves might improve the cellular physiology process. New miRNA, as well as new target genes were found to be differentially expressed following exposure to physiological concentrations of vitamin D. Further investigation is needed to study the specific role of these miRNA, including their target genes and the related molecular and biological pathways. These findings might in the future contribute to understanding the role of vitamin D in treating patients with DM, including detecting new miRNA markers.

## Competing interests

The authors declare that they have no competing interests.

## Authors’ contributions

The individual contributions of each co-author are detailed below: TZG and SB conception and design of research; TZG and JG performed experiments; TZG and MPC analyzed data, interpreted results of experiments and prepared figures; TZG and JG drafted manuscript; TZG, SB, EG and JB edited and revised manuscript; TZG and SB approved final version of manuscript. All authors read and approved the final manuscript.
